# Digitale Gesundheitsanwendungen – was sollten wir als Rheumatolog:innen wissen

**DOI:** 10.1007/s00393-024-01570-3

**Published:** 2024-09-10

**Authors:** Johannes Knitza, Martin Krusche, Gamal Chehab, Christof Specker, Jutta G. Richter

**Affiliations:** 1 Institut für Digitale Medizin, Philipps Universität Marburg und Universitätsklinikum Gießen und Marburg, Baldingerstraße, 35042 Marburg, Deutschland; 2 III. Medizinische Klinik und Poliklinik für Nephrologie, Rheumatologie und Endokrinologie, Universitätsklinikum Hamburg-Eppendorf, Martinistr. 52, 20251 Hamburg, Deutschland; 3 Klinik für Rheumatologie, Medizinische Fakultät, Universitätsklinikum Düsseldorf, Heinrich-Heine-Universität Düsseldorf, Moorenstr. 5, 40225 Düsseldorf, Deutschland; 4 Hiller Forschungszentrum Rheumatologie, Medizinische Fakultät, Universitätsklinikum Düsseldorf, Heinrich-Heine-Universität Düsseldorf, Moorenstr. 5, 40225 Düsseldorf, Deutschland; 5 Klinik für Rheumatologie & Klinische Immunologie, KEM | Evang. Kliniken Essen-Mitte gGmbH, Pattbergstr. 1–3, 45239 Essen, Deutschland

**Keywords:** DiGA, Digitale Gesundheitsanwendung, Digitalisierung, Rheumatologie, E‑Health, DHA, Digital health applications, Digitalization, Rheumatology, E‑Health

## Abstract

Digitale Gesundheitsanwendungen (DiGA) revolutionieren die Patientenversorgung durch verbesserten Zugang zu evidenzbasierter Therapie und fördern aktives Selbstmanagement. Die kontinuierlich wachsende Anzahl an DiGA ermöglicht es Patient:innen, durch digitale Unterstützung eigenständiger zu handeln. Die budgetneutrale Verordnung und Kostenübernahme durch gesetzliche Krankenkassen senken finanzielle Barrieren für Behandler:innen und Patient:innen. Erste Studien belegen, dass DiGA erfolgreich zur Behandlung von Komorbiditäten und rheumatischen Erkrankungen eingesetzt werden können. Mehrere DiGA für entzündlich-rheumatische Erkrankungen befinden sich in fortgeschrittener Entwicklung. Die Identifikation geeigneter Patient:innen und die Unterstützung durch Shared Decision Making (SDM) sind entscheidend für die erfolgreiche Implementierung. Herausforderungen bestehen weiterhin in der Adhärenz und Akzeptanz der Anwendungen. Dieser Artikel bietet einen Überblick über die Verordnung in der Praxis, erste Daten und Erfahrungen aus der rheumatologischen Versorgungsrealität und berichtet über aktuelle Entwicklungen.

## Abstract (en)

Digital health applications (DHAs) are revolutionising patient care by improving access to evidence-based therapy and promoting active self-management. The continuously growing number of DHAs enables patients to act more independently through digital support. The budget-neutral prescription and cost coverage by statutory health insurance companies reduce financial barriers for practitioners and patients. Initial studies show that DHAs can be used successfully to treat comorbidities and rheumatic diseases. Several DHAs for inflammatory rheumatic diseases are at an advanced stage of development. The identification of suitable patients and support through shared decision making are crucial for successful implementation. Challenges remain in adherence and acceptance of the applications. This article provides an overview of prescription in clinical routine, initial data and experiences from the reality of rheumatology care, and reports on current developments.

Durch das Digitale-Versorgung-Gesetz (DVG) können Ärzt:innen und Psychotherapeut:innnen seit September 2020 digitale Gesundheitsanwendungen (DiGA) verordnen. Diese ermöglichen einen zeit- und ortsunabhängigen Zugang zu standardisierter und evidenzbasierter Behandlungsunterstützung. Diese digitale Unterstützung könnte, gerade in Zeiten des Fachärzt:innenmangels, zu einer besseren rheumatologischen Versorgung beitragen. Die DiGA sollen Patient:innen befähigen und motivieren, ihre Erkankung(en) gemäß den Empfehlungen von Fachgesellschaften [1] aktiver selbstständig zu managen. Durch die gesetzliche Regelung wurden mehrere bisherige Barrieren [2], wie unzureichende Evidenz, Sicherheitslücken und fehlende Kostenerstattung konventioneller Gesundheits-Apps überwunden. Die DiGA-Verordnung nimmt kontinuierlich zu, und zwischen 2020 und 2023 wurden laut Daten des Spitzenverbands der Gesetzlichen Krankenversicherung (GKV) 374.000 DiGA-Rezepte eingelöst, siehe Abb. 1.

**Abb. 1:**
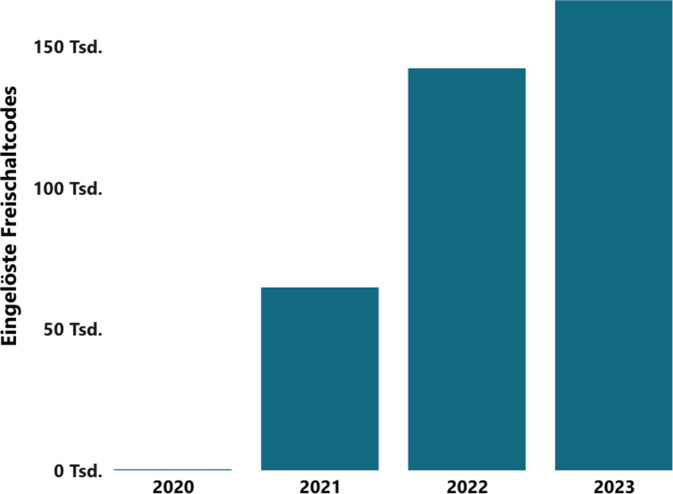
Eingelöste DiGA-Freischaltcodes zwischen 2020 und 2023 laut GKV-Spitzenverband (*DiGA*: Digitale Gesundheitsanwendungen, *GKV*: Gesetzliche Krankenversicherung; [3])

## DiGA-Definition

Digitale Gesundheitsanwendungen sind softwarebasierte Anwendungen, die gesundheitsbezogene Dienstleistungen erbringen. Dabei handelt es sich um Medizinprodukte mit niedriger Risikoklasse (I und IIa), deren Ziele in den gesundheitsbezogenen Zweckbestimmungen festgehalten sind. Typischerweise beinhalten sie Funktionen wie Gesundheits‑/Krankheitsüberwachung, Diagnose- und Therapieunterstützung, Präventionsmaßnahmen oder Gesundheitsinformationen. Diese Anwendungen können auf verschiedenen Plattformen wie Smartphones, Tablets oder Computern ausgeführt werden und werden als installierbare (sog. native App) oder als webbasierte Anwendung (sog. Web App) zur Verfügung gestellt. Dabei werden zunehmend auch moderne IT-Technologien wie künstliche Intelligenz, maschinelles Lernen und zusätzliche Hardware genutzt, um diese Dienste bereitzustellen. Beispielsweise gibt es auch DiGA (z. B. Invirto), die in Kombination mit einer zur Verfügung gestellten Virtual-Reality-Brille zur Behandlung von Agoraphobie eingesetzt werden. Kürzlich erschien eine Studie, die den Mehrwert einer solchen digitalen Therapie mit Virtual-Reality-Brille für Fibromyalgie-Patient:innen demonstrieren konnte [4].

## DiGA-Zulassung

Um als DiGA zugelassen zu werden, müssen Hersteller einen Antragsprozess beim Bundesinstitut für Arzneimittel und Medizinprodukte (BfArM) durchlaufen, der eine detaillierte Bewertung der DiGA-Eigenschaften und ihrer Eignung für den Einsatz umfasst. Dieser Prozess beinhaltet die Einreichung umfangreicher Unterlagen, einschließlich klinischer Studien, die einen positiven Versorgungseffekt demonstrieren [5], Sicherheitsnachweise und Qualitätsbewertungen.

Das BfArM bewertet DiGA anhand verschiedener Kriterien, darunter:

- **Sicherheit:** Die Anwendung muss nachweislich sicher sein und potenzielle Risiken für die Nutzer:innen minimieren.
- **Wirksamkeit:** Es muss wissenschaftliche Evidenz dafür vorliegen, dass die Anwendung die beabsichtigten Gesundheitsziele erreicht.
- **Qualität:** Die Anwendung muss qualitativ hochwertig sein und den geltenden Standards für medizinische Software (MDR) entsprechen.
- **Datenschutz und Datensicherheit:** Es müssen entsprechende Mechanismen implementiert sein, um die Privatsphäre und den Datenschutz der Nutzer zu gewährleisten.
- **Nutzerfreundlichkeit:** Die Anwendung sollte benutzerfreundlich gestaltet sein und eine einfache Bedienung ermöglichen.

Weitere Anforderungen werden beispielsweise an Interoperabilität, Robustheit, Verbraucherschutz und die Unterstützung der Leistungserbringenden gestellt. Das BfArM hat einen DiGA-Leitfaden [6] entwickelt um Hersteller, Leistungserbringer und Anwender zu unterstützen.

Man unterscheidet zwischen einer dauerhaften und einer vorläufigen Zulassung. Bei einer vorläufigen Zulassung muss mindestens ein Evidenzkonzept und ein klinischer Prüfplan vorliegen. Vorteil der vorläufigen Zulassung ist eine direkte Erstattung, während die Evidenz noch mittels Zulassungsstudie generiert wird. Die Hersteller müssen innerhalb eines Zeitraums von 12 Monaten einen Nachweis für einen positiven Versorgungseffekt erbringen. Das BfArM prüft dann innerhalb von drei Monaten die vorgelegten Studiendaten und trifft eine Entscheidung. Wenn kein Studienergebnis eingereicht oder der Antrag abgelehnt wird, wird die DiGA wieder aus dem DiGA-Verzeichnis beim BfArM gestrichen. Der DiGA-Hersteller kann dann frühestens 12 Monate nach dem ablehnenden Bescheid einen neuen Antrag einreichen.

## DiGA-Verzeichnis

Das DiGA-Verzeichnis (https://diga.bfarm.de) des BfArM ermöglicht einen schnellen und aktuellen Überblick über alle verordnungsfähigen DiGA. Das Verzeichnis ist ähnlich zu konventionellen App-Stores aufgebaut und man kann mit mehreren Filtern arbeiten. Aktuell gibt es 57 zugelassene und erstattungsfähige DiGA in Deutschland auf dem Markt (Stand 10.05.2024). Davon sind 35 dauerhaft zugelassen und 22 haben einen vorläufigen Status. Die häufigste Kategorie ist Psyche mit 17 dauerhaft zugelassenen und 9 vorläufig zugelassenen DiGA. Acht DiGA zielen auf Erkrankungen, die die Muskeln, Knochen und Gelenke betreffen, wobei hier überwiegend degenerative Erkrankungen wie z. B. Gonarthrose oder Osteochondrose der Wirbelsäule im Fokus stehen.

## How to DiGA – DiGA-Auswahl und Verordnung

Digitale Therapiemöglichkeiten werden explizit zur Unterstützung der Behandlung von rheumatischen Patient:innen empfohlen [1, 7, 8]. Die Nutzung digitaler therapeutischer Tools durch Patient:innen und Rheumatolog:innen hat in den letzten Jahren zugenommen [9], jedoch wurde das Potenzial der DiGA bislang nur unzureichend in der Rheumatologie ausgeschöpft [10].

Die Identifikation geeigneter Patient:innen für eine DiGA-Nutzung ist von besonderer Bedeutung, da mangelnde IT-Kenntnisse oder fehlende Aufgeschlossenheit Hürden für eine effektive Nutzung darstellen können und zu einer mangelnden Adhärenz und unnötigen Kosten führen können. Umso wichtiger ist es, in einem ersten Schritt zusammen mit Patient:innen in Form eines Shared Decision Making (SDM) die individuelle Praktikabilität zu erörtern und nachfolgend eine geeignete DiGA zu identifizieren. Die letzte Auswertung des GKV-Spitzenverbands im September 2023 zeigte, dass die Mehrheit (71 %) der 374.377 verordneten DiGA Frauen verordnet wurden und das mittlere Alter bei 45 Jahren lag [11]. Ähnliche Daten liefert auch der Report der Techniker Krankenkasse [15].

**Abb. 2:**
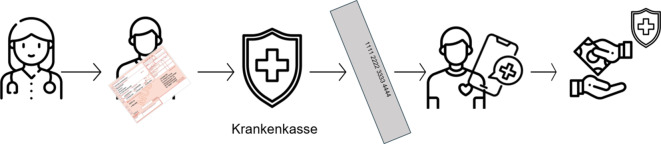
Klassische DiGA-Verordnung und Aktivierung. Ärzt:innen und Psychotherapeut:innen können mittels Rezept (Muster 16) und Pharmazentralnummer (PZN) digitale Gesundheitsanwendungen (DiGA) wie konventionelle Arzneimittel verordnen. Patient:innen reichen dieses Rezept bei ihrer Krankenkasse ein. Die Krankenkassen müssen dann innerhalb von 48 h Patient:innen einen Freischaltcode zukommen lassen. Mit diesem Code können Patient:innen dann die DiGA für den vorhergesehenen Zeitraum nutzen. Hersteller rechnen direkt mit den Krankenkassen ab. Alternativ können Patient:innen auch direkt bei ihrer Krankenkasse eine DiGA beantragen

Eine Verordnung (Abb. 2) erfolgt in der Regel für einen Zeitraum von 90 Tagen. Die Verordnung mehrerer DiGA ist prinzipiell möglich, jedoch zeigt die Erfahrung der Autor:innen, dass eine adäquate Nutzung in der Praxis durch den nicht unerheblichen Aufwand für Patient:innen unrealistisch ist. Ob es zu „Interaktionen“ zwischen mehreren verordneten DiGA kommen kann, ist aktuell noch unklar [12]. Für eine In-label-Verordnung muss bei den Patient:innen eine entsprechende, dokumentierte, für die DiGA zugelassene Indikation mittels ICD-10 Code vorliegen, und es dürfen keine der gelisteten (s. BfArM-DiGA-Verzeichnis) Kontraindikationen vorliegen (DiGA-spezifisch, z. B. kognitive oder physische Einschränkungen, welche die empfohlene Anwendung nicht zulassen; Tumorschmerzen; Suizidalität). Patient:innen sollten auch über die Funktionsweise der DiGA informiert und in deren Bedienung angeleitet werden. Hier bietet es sich an, Patient:innen Infomaterial wie Flyer mit Support-Hotline oder QR-Codes zur Produkt-Website der Hersteller zur Verfügung zu stellen. Zum Großteil können Patient:innen die DiGA auch ohne Aktivierungscode bereits für einige Tage testen und Hersteller bei Fragen kontaktieren. Die Hersteller sind gesetzlich verpflichtet, Anfragen von Patient:innen innerhalb von 24 h zu beantworten. Die DiGA können dann mittels Rezept (Muster 16) und Pharmazentralnummer (PZN) und DiGA-Namen wie konventionelle Arzneimittel verordnet werden. Klassischerweise reichen Patient:innen das DiGA-Rezept selbst bei ihrer Krankenkasse ein. Alternativ können Patient:innen das Rezept auch über Rezept-Service-Anbieter und teilweise auch nach Registrierung mittels der DiGA der Krankenkasse übermitteln. Die GKV-Krankenkassen müssen dann Patient:innen innerhalb von 48 h einen Freischaltcode zukommen lassen. Mit diesem Code können Patient:innen dann die DiGA für den vorhergesehenen Zeitraum nutzen. Die Erstattung durch private Krankenversicherer ist noch nicht einheitlich geregelt. Es ist ratsam, sich mit der Funktionsweise von DiGA auch vonseiten der Verordnenden vertraut zu machen. Die Deutsche Gesellschaft für Innere Medizin (DGIM) hat einen Kriterienkatalog für kurze DiGA-Erklärvideos erstellt [13]. Noch besser ist es, mittels DiGA-Testzugängen die DiGA selbst zu testen. DiGA-Testzugänge können beim jeweiligen Hersteller angefragt werden. Abb. 2 beschreibt den klassischen Verordnungsprozess. Alternativ können DiGA auch direkt von Patient:innen bei der Krankenkasse beantragt werden.

## DiGA-Vergütung und Kosten

Die Verordnung von DiGA ist für Verordner:innen budgetneutral. Für die Verordnung einer DiGA ist seit 2023 keine Vergütung mehr möglich, die Erstverordnung einer DiGA ist Bestandteil der Versicherten- und Grundpauschalen. Jedoch ist bei einzelnen DiGA eine Vergütung für Verlaufskontrollen und Auswertungen abgebildet. Für vorläufig in das DiGA-Verzeichnis aufgenommene DiGA kann von Ärzt:innen und Psychotherapeut:innen mit Stand 03/2024 die Pauschale 86.700 (7,12 €) für die Verlaufskontrolle und Auswertung von DiGA abgerechnet werden [14]. Für die einzelnen dauerhaft zugelassenen DiGA stehen unterschiedliche Gebührenordnungspositionen nach dem einheitlichen Bewertungsmaßstab (EBM) zur Verfügung [14]. Laut Kassenärztlicher Bundesvereinigung (KBV) ist „auch bei der DiGA-Verordnung das Wirtschaftlichkeitsgebot zu beachten, wonach die Leistung ausreichend, zweckmäßig und wirtschaftlich sein muss (§ 12 SGB V)“ [14].

Die Preisfestsetzung von DiGA folgt dem Vorbild des Arzneimittelmarktneuordnungsgesetzes (AMNOG). Für die ersten 12 Monate erfolgt die Preisfestsetzung durch den Hersteller, danach wird der finale Preis zwischen Hersteller und GKV-Spitzenverband verhandelt. Die Differenz ist rückwirkend auszugleichen. Die DiGA-Kosten variieren derzeit zwischen 119,00 € (Mawendo) und 2077,40 € (levidex), wobei die durchschnittlichen Preise in den letzten Jahren eher gestiegen sind (2020: 418 €; 2023:628 €) [15]. Generell werden DiGA günstiger, desto mehr DiGA bereits für die jeweilige Indikation gelistet sind.

Die aktuellen Preise pro DiGA stellt Tab. 1 dar. Vonseiten der Kostenträger wird wiederholt die wirtschaftliche Bedeutung der „Nichtnutzer“ verordneter DiGA kritisiert und neue Erstattungsmodelle diskutiert, wie z. B. ein 14-tägiger Probezeitraum. Ebenfalls diskutiert werden Pay-for-Performance-Modelle [16], wobei eine Erstattung nur bei nachgewiesener Nutzung oder sogar nachgewiesenem Therapieerfolg erfolgt.

**Tab. 1:** Verordnungen von DiGA bei der Barmer Krankenkasse nach Anwendungskategorien 2022 [20]

Kategorie	Anzahl der DiGA	Anzahl der Verordnungen	Anzahl der Genehmigungen	Prozentsatz der Erstattung	Erstattungsbetrag (Euro)
Bewegungsapparat	4	45.216	33.452	74,9	263
Adipositas	2	41.133	35.702	90,8	473
Tinnitus	2	34.259	27.577	81,4	231
Depressionen	3	26.910	20.888	78,9	326
Angststörungen	8	18.113	14.007	79,5	479
Schlafstörungen	1	16.886	14.489	86,5	226
Erektionsstörungen	1	7611	4590	81,8	651
Migräne	2	6398	4728	82,3	281
Endometriose	1	5428	4344	95,2	599
Krebs	4	4287	3548	87,8	545
Aphasie	1	3923	3221	89,1	488
Stress, Burn-out	1	3668	2621	74,3	454
Rauchen	1	3527	2462	71,4	301
Schmerzen	1	3130	2440	78,1	601
Reizdarm	1	2758	2478	93,1	718
Multiple Sklerose	1	2347	1911	82,2	417
Sonstige	4	2039	1344	77,2	517
Diabetes	3	864	713	82,5	518

Ob eine DiGA-Folgeverordnung Sinn macht, sollte individuell geprüft werden. Je nach DiGA sind primäre Endpunkte in Zulassungsstudien zum Teil auch erst nach 6 Monaten erhoben worden. Bisherige Erfahrungen legen jedoch nahe, dass die DiGA-Adhärenz mit der Zeit eher abnimmt [17–19]. Wichtig ist dabei, dass DiGA i. d. R. nicht generell jeden Tag genutzt werden müssen, um einen Therapieeffekt zu erzielen [19].

## DiGA für entzündlich-rheumatische Erkrankungen?

Bislang ist keine DiGA zur Behandlungsergänzung entzündlich-rheumatischer Erkrankungen zugelassen, jedoch ist eine Vielzahl in Entwicklung und erste positive Zulassungsstudien sind bereits abgeschlossen [21]. Bisherige rheumatologische DiGA-Anwärter (vgl. Tab. 2) adressieren vor allem häufige entzündlich-rheumatische Erkrankungen und basieren auf etablierten Therapieprinzipien wie der kognitiven Verhaltenstherapie, Bewegungstherapie bzw. vielfältigen Vorschlägen zur Lebensstilmodifikation. Erste publizierte Ergebnisse sind vielversprechend und lassen auf baldige Zulassungen hoffen.

**Tab. 2:** Überblick über zertifizierte Medizinprodukte, für die eine Zulassung als digitale Gesundheitsanwendung für entzündlich-rheumatische Erkrankungen beantragt wurde bzw. wird

Produktname	Indikationen	Plattform	Hauptfunktion	Assoziierte Studien	Auswahl bisher publizierter positiver Versorgungseffekte
Axia	axSpA	Native App	Bewegungstherapie	DRKS00033783	Steigerung der Übungsfrequenz, Steigerung des Bewegungsumfang, Schmerzreduktion, gesteigertes krankheitsspezifisches Wissen [22]
Mida Rheuma	axSpA	Native App	Gesundheitscoach	DRKS00025966	Steigerung der Lebensqualität, Reduktion der Krankheitsaktivität [23]
Reclarit	axSpA,PsA,RA,SLE	Web App	Kognitive Verhaltenstherapie	DRKS00025256	Steigerung der Lebensqualität, Reduktion von Fatigue, Depression und Angst [21]
RheCORD	PsA,RA,SLE	Native App	Gesundheitscoach	DRKS00032185	Keine
Vila RaVie	PsA,RA,SLE	Web App	Kognitive Verhaltenstherapie	DRKS00032862	Keine

*axSpA* Axiale Spondylarthritis, *PsA* Psoriasisarthritis, *RA* Rheumatoide Arthritis, *SLE* Systemischer Lupus erythematodes

**Abb. 3:**
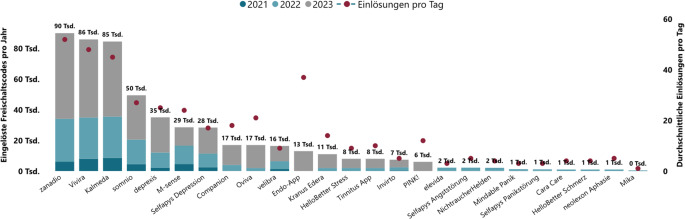
Kumulierte eingelöste DiGA-Freischaltcodes pro Jahr (2021–2023) und Einlösungen pro Tag nach Jahresberichten des GKV-Spitzenverbands [3]

Das optimale Management entzündlich-rheumatischer (System‑)Erkrankungen erfordert auch eine effektive Behandlung von Begleit- und Folgeerkrankungen. Bereits jetzt kann man häufige Komorbiditäten rheumatologischer Patient:innen mit DiGA adressieren. Die meisten DiGA bieten psychologische Hilfe, sei es bei der Bewältigung von chronischen Schmerzen, Fibromyalgie oder klassischen Depressionen [24]. Patient:innen können auch bei der Reduktion kardiovaskulärer Risikofaktoren wie Gewichtsreduktion, Hypertonus, Diabetes und der Raucherentwöhnung unterstützt werden. Ein anderer therapeutischer Ansatz ist die Anleitung für körperliches Training zu Hause, bspw. zur Behandlung unspezifischer Rückenschmerzen und Gonarthrose. Zu den bislang am häufigsten verordneten DiGA (Abb. 3) gehören zanadio (Gewichtsreduktion), Vivira (Reduktion von Rückenschmerzen) und Kalmeda (Tinnitus).

## Erste Real-World-Evidence aus der rheumatologischen Routine

Eine erste Pilotstudie (*n* = 48) untersuchte, welche DiGA bei rheumatologischen Patient:innen in einer Hochschulambulanz verordnet wurden, wie die Adhärenz und der empfundene Nutzen von Patient:innen war [18]. Das mediane Alter lag bei 50 Jahren, 60 % der Patient:innen waren weiblich. Die meisten Patient:innen hatten eine axiale Spondylarthritis (44 %). Die häufigsten DiGA-Indikationen waren „unspezifische Rückenschmerzen“ (46 %) und eine chronische Schmerzstörung mit somatischen und psychischen Faktoren. Nur 51 % der Patient:innen gab an, die DiGA überhaupt genutzt zu haben und nur 13 % vollumfänglich über 3 Monate. Es gaben 40 % der Patient:innen, die eine DiGA nutzten, eine Beschwerdebesserung an. In der Analyse zeigten sich Alters- und Geschlechtsunterschiede. Patient:innen mit geringer Nutzung und geringer Beschwerdebesserung waren signifikant älter und zumindest numerisch häufiger männlich. Interessanterweise nutzten alle Patient:innen, die eine Besserung angaben, eine Rückenschmerz-DiGA. Die Studie zeigt in Einklang mit der Literatur, dass mangelnde Adhärenz eine wesentliche Implementierungsbarriere digitaler Medizinprodukte in der Rheumatologie darstellt [25]. Sie verdeutlicht jedoch auch, dass bereits jetzt Behandlungserfolge mit DiGA in der Rheumatologie möglich sind. Das Potenzial für Ärzt:innen, den individuellen Behandlungserfolg über die Verordnung einer DiGA positiv zu beeinflussen, sollte nicht unterschätzt werden [17].

In Anbetracht der zunehmenden Versorgungslücken stellen DiGA ein vielversprechendes zusätzliches Hilfsmittel dar, um Wartezeiten zu verkürzen und Patient:innen aktiver in die eigene Behandlung einzubinden [26]. Ähnliche Ansätze werden zunehmend in Ländern wie Frankreich mit dem Prise-en-Charge-Anticipée(PECAN)-Programm und Belgien mit mhealthBelgium [27] implementiert.

## Fazit für die Praxis

- DiGA bieten eine ergänzende budgetneutrale Therapiemöglichkeit, um das Selbstmanagement von Patient:innen zu fördern.
- DiGA ermöglichen einen schnellen und skalierbaren Zugang zu zeit- und ortsunabhängiger evidenzbasierter Behandlungsergänzung.
- Bereits jetzt lassen sich Komorbiditäten von entzündlich-rheumatischen Patient:innen mit DiGA adressieren.
- Mehrere DiGA für entzündlich rheumatische Erkrankungen befinden sich aktuell in der Entwicklung bzw. wurden bereits positiv in Studien evaluiert und der Zulassungsprozess initiiert.

## Data Availability

Die Daten werden bei begründeter Anfrage zur Verfügung gestellt.

## References

[1] Nikiphorou E, Santos EJF, Marques A, Böhm P, Bijlsma JW, Daien CI et al (2021) 2021 EULAR recommendations for the implementation of self-management strategies in patients with inflammatory arthritis. Ann Rheum Dis (https://ard.bmj.com/content/early/2021/06/13/annrheumdis-2021-220249) 10.1136/annrheumdis-2021-220249 PMC8458093 33962964

[2] Knitza J, Tascilar K, Messner EM, Meyer M, Vossen D, Pulla A et al (2019) German mobile Apps in rheumatology: review and analysis using the mobile application rating scale (MARS). JMIR Mhealth Uhealth 7(8):e14991 31381501 10.2196/14991 PMC6699116

[3] fbeta (2024) DiGA-Analyzer. https://fbeta.de/diga-analyzer/

[4] Christensen SWM, Almsborg H, Vain TS, Vaegter HB (2023) The effect of virtual reality on cold pain sensitivity in patients with fibromyalgia and pain-free individuals: a randomized crossover study. Games Health J 12(4):295–301 36454199 10.1089/g4h.2022.0138

[5] König IR, Mittermaier M, Sina C, Raspe M, Stais P, Gamstätter T et al (2022) Evidence of positive care effects by digital health apps-methodological challenges and approaches. Inn Med 63(12):1298–1306 10.1007/s00108-022-01429-2 36279007

[6] BfArM (2024) DiGA-Leitfaden. https://www.bfarm.de/SharedDocs/Downloads/DE/Medizinprodukte/diga_leitfaden.html. Zugegriffen: 9. Juni 2024

[7] Knitza J, Callhoff J, Chehab G, Hueber A, Kiltz U, Kleyer A et al (2020) Position paper of the commission on digital rheumatology of the German Society of Rheumatology: tasks, targets and perspectives for a modern rheumatology. Z Rheumatol 79(6):562–569 32651681 10.1007/s00393-020-00834-y

[8] de Thurah A, Bosch P, Marques A, Meissner Y, Mukhtyar CB, Knitza J et al (2022) 2022 EULAR points to consider for remote care in rheumatic and musculoskeletal diseases. Ann Rheum Dis 10.1136/annrheumdis-2022-222341 35470160

[9] Kernder A, Morf H, Klemm P, Vossen D, Haase I, Mucke J et al (2021) Digital rheumatology in the era of COVID-19: results of a national patient and physician survey. RMD Open 7(1):e1548 33622673 10.1136/rmdopen-2020-001548 PMC7907631

[10] Richter JG, Chehab G, Stachwitz P, Hagen J, Larsen D, Knitza J et al (2022) One year of digital health applications (DiGA) in Germany – Rheumatologists’ perspectives. Front Med 9:1000668 10.3389/fmed.2022.1000668 PMC9640713 36388899

[11] GKV-Spitzenverband. Bericht des GKV-Spitzenverbandes über die Inanspruchnahme und Entwicklung der Versorgung mit Digitalen Gesundheitsanwendungen (DiGA-Bericht) gemäß § 33a Absatz 6 SGB V [Internet]. Bericht des GKV-Spitzenverbandes über die Inanspruchnahme und Entwicklung der Versorgung mit Digitalen Gesundheitsanwendungen. [zitiert 8. Juni 2024]. Verfügbar unter: https://www.gkv-spitzenverband.de/media/dokumente/krankenversicherung_1/telematik/digitales/2023_DiGA_Bericht_GKV-Spitzenverband.pdf

[12] Mittermaier M, Sina C, Richter JG, Raspe M, Stais P, Vehreschild J et al (2022) Practical use of digital health applications (DiGA) in internal medicine. Internist 63(3):245–254 35037948 10.1007/s00108-022-01264-5

[13] AG Digitale Gesundheitsanwendungen (DiGA) KI in Leitlinien der Deutschen Gesellschaft für Innere Medizin e. V. Kriterienkatalog Erklärvideos für digitale Gesundheitsanwendungen [Internet]. Kriterienkatalog Erklärvideos für digitale Gesundheitsanwendungen. https://www.dgim.de/fileadmin/user_upload/PDF/UEber_uns/Gremien/Digitale_Transformation/20230412_FINAL_Kriterienkatalog_Erklaervideos.pdf. Zugegriffen: 3. Juli 2024

[14] Kassenärztliche Bundesvereinigung Digitale Gesundheitsanwendungen: Hinweise Zur Verordnung, Abrechnung Und Vergütung. https://www.kbv.de/media/sp/PraxisInfo_Digitale_Gesundheitsanwendungen.pdf. Zugegriffen: 9. Juni 2024

[15] TK DiGA-Report II 2024. https://www.tk.de/presse/themen/digitale-gesundheit/digitaler-fortschritt/diga-report-2-2024-2125138. Zugegriffen: 9. Juni 2024

[16] Knitza J, Muehlensiepen F, Kuhn S (2023) Digital health applications: toward a lifecycle and pay-for-performance approach. Mayo Clin Proc Digit Health 1(3):393–394 10.1016/j.mcpdig.2023.07.001 PMC11975719 40206613

[17] Dahlhausen F, Zinner M, Bieske L, Ehlers JP, Boehme P, Fehring L (2022) There’s an app for that, but nobody’s using it: Insights on improving patient access and adherence to digital therapeutics in Germany. Digit Health 8:20552076221104672 35811758 10.1177/20552076221104672 PMC9260569

[18] Labinsky H, Gupta L, Raimondo MG, Schett G, Knitza J (2023) Real-world usage of digital health applications (DiGA) in rheumatology: results from a German patient survey. Rheumatol Int 43(4):713–719 36543961 10.1007/s00296-022-05261-7 PMC9770561

[19] Priebe JA, Kerkemeyer L, Haas KK, Achtert K, Moreno Sanchez LF, Stockert P et al (2024) Medical app treatment of non-specific low back pain in the 12-month cluster-randomized controlled trial rise-uP: where clinical superiority meets cost savings. J Pain Res 17:2239–2255 38952994 10.2147/JPR.S473250 PMC11215667

[20] Grobe T, Weller L, Braun A, Szecsenyi J BARMER Arztreport 2024. BARMER, Berlin

[21] Betz LT, Jacob GA, Knitza J, Koehm M, Behrens F (2024) Efficacy of a cognitive-behavioral digital therapeutic on psychosocial outcomes in rheumatoid arthritis: randomized controlled trial. Npj Ment Health Res 3:41 39227501 10.1038/s44184-024-00085-8 PMC11371912

[22] Strunz PP, Le Maire M, Heusinger T, Klein J, Labinsky H, Fleischer A et al (2024) The exercise-app Axia for axial spondyloarthritis enhances the home-based exercise frequency in axial spondyloarthritis patients—A cross-sectional survey. Rheumatol Int 10.1007/s00296-024-05600-w PMC11108939 38683351

[23] Fedkov D, Berghofen A, Weiss C, Peine C, Lang F, Knitza J et al (2022) Efficacy and safety of a mobile app intervention in patients with inflammatory arthritis: a prospective pilot study. Rheumatol Int 42(12):2177–2190 36112186 10.1007/s00296-022-05175-4 PMC9483251

[24] Haaf R, Vock P, Wächtershäuser N, Correll CU, Köhler S, Klein JP (2024) Efficacy of internet-based interventions for depression available in Germany—A systematic review and meta-analysis. Nervenarzt 95(3):206–215 38260995 10.1007/s00115-023-01587-0 PMC10914865

[25] Druce KL, Dixon WG, McBeth J (2019) Maximizing engagement in mobile health studies: lessons learned and future directions. Rheum Dis Clin North Am 45(2):159–172 30952390 10.1016/j.rdc.2019.01.004 PMC6483978

[26] Schmidt L, Pawlitzki M, Renard BY, Meuth SG, Masanneck L (2024) The three-year evolution of Germany’s digital therapeutics reimbursement program and its path forward. NPJ Digit Med 7(1):139 38789620 10.1038/s41746-024-01137-1 PMC11126413

[27] Lievevrouw E, Marelli L, Van Hoyweghen I (2024) Weaving EU digital health policy into national healthcare practices. The making of a reimbursement standard for digital health technologies in Belgium. Soc Sci Med 346:116620 38479265 10.1016/j.socscimed.2024.116620

